# Application of an in situ CO_2_–bicarbonate system under nitrogen depletion to improve photosynthetic biomass and starch production and regulate amylose accumulation in a marine green microalga *Tetraselmis subcordiformis*

**DOI:** 10.1186/s13068-019-1523-7

**Published:** 2019-07-16

**Authors:** Man Qi, Changhong Yao, Binhuan Sun, Xupeng Cao, Qiang Fei, Bobo Liang, Wenyi Ran, Qi Xiang, Yongkui Zhang, Xianqiu Lan

**Affiliations:** 1 Department of Pharmaceutical & Biological Engineering, School of Chemical Engineering, Sichuan University, Chengdu, 610065 Sichuan China; 2 State Key Laboratory of Catalysis, Dalian Institute of Chemical Physics, Chinese Academy of Sciences, Dalian, 116023 Liaoning China; 3 Division of Solar Energy, Dalian National Laboratory of Clean Energy, Dalian, 116023 Liaoning China; 4 Biotechnology Department, Dalian Institute of Chemical Physics, Chinese Academy of Sciences, Dalian, 116023 Liaoning China; 5 School of Chemical Engineering and Technology, Xi’an Jiaotong University, Xi’an, 710049 Shaanxi China

**Keywords:** Starch, Nitrogen depletion, pH, Bicarbonate, Amylose, *Tetraselmis subcordiformis*

## Abstract

**Background:**

Microalgal starch is regarded as a promising alternative to crop-based starch for biorefinery such as the production of biofuels and bio-based chemicals. The single or separate use of inorganic carbon source, e.g., CO_2_ and NaHCO_3_, caused aberrant pH, which restricts the biomass and starch production. The present study applied an in situ CO_2_–NaHCO_3_ system to regulate photosynthetic biomass and starch production along with starch quality in a marine green microalga *Tetraselmis subcordiformis* under nitrogen-depletion (−N) and nitrogen-limitation (±N) conditions.

**Results:**

The CO_2_ (2%)–NaHCO_3_ (1 g L^−1^) system stabilized the pH at 7.7 in the −N cultivation, under which the optimal biomass and starch accumulation were achieved. The biomass and starch productivity under −N were improved by 2.1-fold and 1.7-fold, respectively, with 1 g L^−1^ NaHCO_3_ addition compared with the one without NaHCO_3_ addition. NaHCO_3_ addition alleviated the high-dCO_2_ inhibition caused by the single CO_2_ aeration, and provided sufficient effective carbon source HCO_3_^−^ for the maintenance of adequate photosynthetic efficiency and increase in photoprotection to facilitate the biomass and starch production. The amylose content was also increased by 44% under this CO_2_–bicarbonate system compared to the single use of CO_2_. The highest starch productivity of 0.73 g L^−1^ day^−1^ under −N cultivation and highest starch concentration of 4.14 g L^−1^ under ±N cultivation were both achieved with the addition of 1 g L^−1^ NaHCO_3_. These levels were comparable to or exceeded the current achievements reported in studies. The addition of 5 g L^−1^ NaHCO_3_ under ±N cultivation led to a production of high-amylose starch (59.3% of total starch), which could be used as a source of functional food.

**Conclusions:**

The in situ CO_2_–NaHCO_3_ system significantly improved the biomass and starch production in *T. subcordiformis*. It could also regulate the starch quality with varied relative amylose content under different cultivation modes for diverse downstream applications that could promote the economic feasibility of microalgal starch-based biofuel production. Adoption of this system in *T. subcordiformis* would facilitate the CO_2_ mitigation couple with its starch-based biorefinery.

**Electronic supplementary material:**

The online version of this article (10.1186/s13068-019-1523-7) contains supplementary material, which is available to authorized users.

## Background

Microalgae, which can photosynthetically fix CO_2_ and produce a variety of compounds (carbohydrate, lipid and protein), are currently considered as sustainable feedstock for biofuel production and as high-value compounds producers due to their high photosynthetic efficiency, fast growth, robust CO_2_ fixation ability, flexible and controllable cultivation modes, and no competition for arable lands [1]. Starch is the primary photosynthetic carbon sink for many microalgae, the existence of which is especially abundant in Chlorophyta [2]. Because the structure of the starch from microalgae resembles that in the higher plants, it is regarded as a promising alternative to crop-based starch for application in the fields of biofuel generation (such as bioethanol, bio-butanol, bio-methane and bio-hydrogen) and bio-based chemicals production [2, 3].

Considerable microalgal starch accumulation with usually more than 50%DW stored intracellularly occurs under stressful conditions such as nutrient deprivation and high irradiance, with nitrogen depletion (−N) or nitrogen limitation (±N) being the most widely studied strategies for the improvement of starch production [2, 4–7]. In general, the −N cultivation, which in essential applies a low cell density and short cultivation time without extra or with very small amounts of nitrogen supply, can enable a relatively high light availability for an individual microalga that tends to facilitate the rapid starch accumulation with high starch productivity and content; in contrast, ±N cultivation employs a limited nitrogen supply for cell growth, which needs longer cultivation time and can get more biomass and improve starch concentration [5]. These two cultivation modes can be combined as a “two-stage” process to incorporate their respective advantages, which can maximize the starch production [8].

Another important factor affecting microalgal biomass and starch production is the carbon supply. In general, microalgae utilize CO_2_ as the direct carbon source for photosynthesis. However, due to the low water solubility, gaseous CO_2_ supply in air cannot meet the desired biomass productivity [9]. Moreover, although the increase in CO_2_ percentage in air during aeration can improve the carbon availability in the medium, the pH will decrease, which could in turn inhibit the microalgal growth [10]. Bicarbonate is another effective carbon source that most microalgae can utilize. It can be converted to CO_2_ via the action of carbonic anhydrase (CA) enzyme and then be fixed via photosynthesis [11]. NaHCO_3_, which has high water solubility and is widely available with a low price, has been recently used to increase carbon supply and improve biomass and lipid/carbohydrate production in several microalgae such as *Tetraselmis suecica* [12], *Chlorella vulgaris* [13], *Scenedesmus* sp. [14] and *Dunaliella salina* V-101 [15]. However, the single use of NaHCO_3_ increased the pH (usually > 10 on the final cultivation day) due to the utilization of HCO_3_^−^ by microalgae that tended to release OH^−^ according to the equilibrium relationship of HCO_3_^−^ + H_2_O ↔ H_2_CO_3_ + OH^−^ and H_2_CO_3_ ↔ CO_2_ + H_2_O, and hence, the biomass production was still limited [16, 17]. Moreover, the starch accumulation could also be influenced by the varied pH environments originated from the different carbon sources used [18, 19]. Therefore, to get an optimized biomass or starch production, suitable supply of carbon source is required to ensure a carbon-abundant environment along with a favorable pH condition.

Traditionally, pH is controlled by adding acid (including CO_2_) or alkali, which usually incorporated a complex online monitoring system [20], making it difficult to be realized in large-scale cultivations especially when large open ponds are used. Recently, Zhu et al. [21] established a recycling culture in which HCO_3_^−^ was first used for microalgal growth followed by CO_2_ neutralization for medium recycle. However, this strategy required a good tolerance of microalgae to high pH (typically > 9) because in essential HCO_3_^−^ was used solely in the cultivation stage. The combined use of CO_2_ and NaHCO_3_, which can construct a CO_2_–NaHCO_3_ buffering system and hence avoid the pH problem of the single use, had recently been demonstrated to enhance the algal growth rate and carbon utilization efficiency as well as lipid production in *Chlorella* [9, 22]. The aeration of CO_2_ will in situ neutralize the OH^−^ derived from the uptake of HCO_3_^−^ and regenerate HCO_3_^−^, and thus, stable and favorable pH can be achieved during the cultivation. However, rare attention has been paid to the effect of CO_2_–NaHCO_3_ system on the starch production under nutrient depletion or limited conditions.

*Tetraselmis subcordiformis* is a marine green microalga that has been demonstrated to accumulate more than 50%DW starch intracellularly under nitrogen deprivation [5, 23]. The present study aimed at further improving the biomass and starch production in this alga via the regulation of pH and effective carbon source using an appropriate in situ CO_2_–NaHCO_3_ system. The starch quality, i.e., the amylose proportion in the total accumulated starch, was also tracked to evaluate the suitability of the starch obtained under different cultivation strategies for the biofuel generation along with additional possible high-value applications that could contribute to the economic feasibility of the whole process.

## Results and discussion

### Biomass production and DIC under nitrogen depletion

**Fig. 1:**
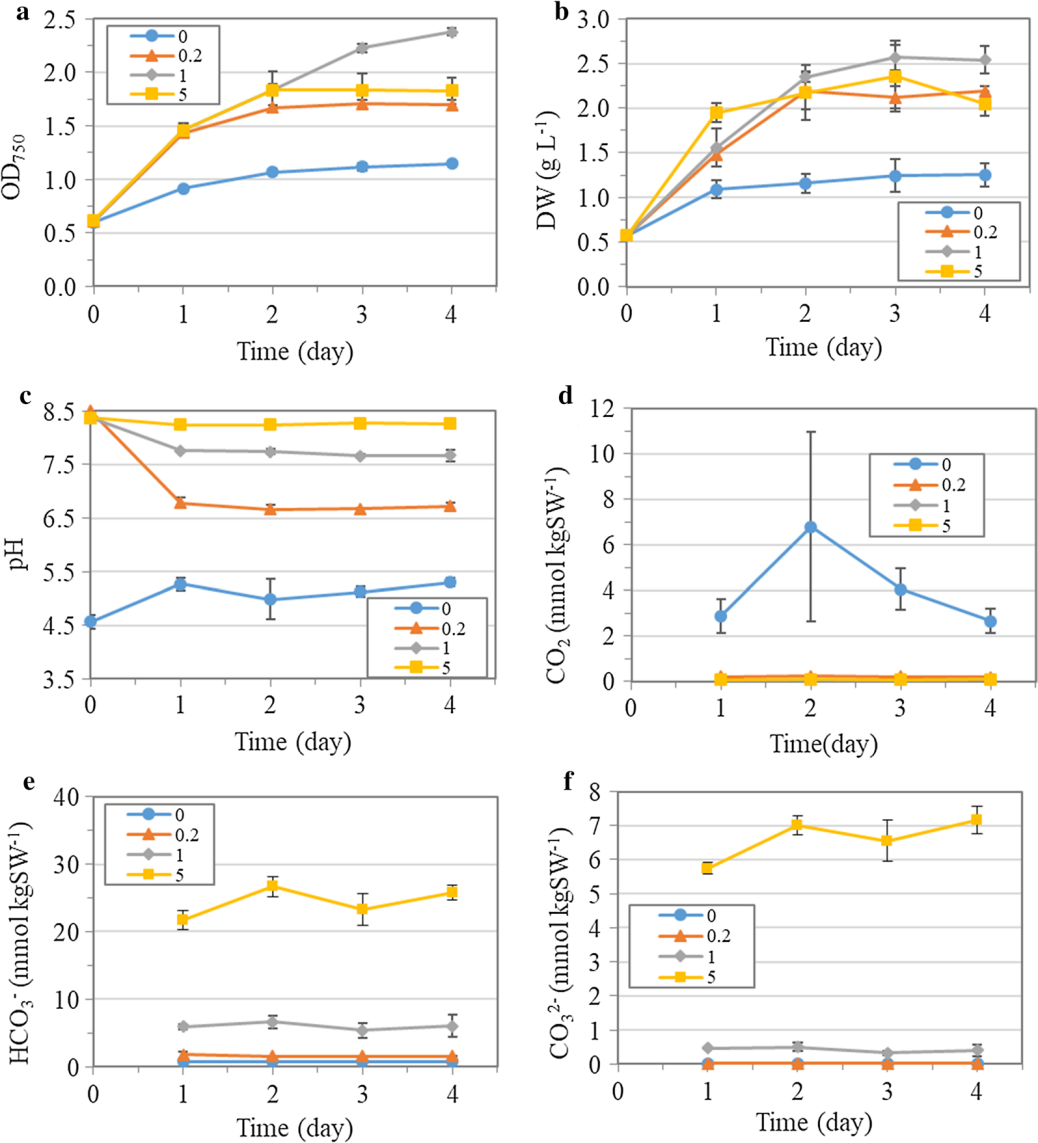
Cell growth (OD_750_, **a**), biomass production (dry weight, **b**), pH variations (**c**), dissolved CO_2_ concentration (dCO_2_, **d**), HCO_3_^−^ concentration (**e**) and CO_3_^2−^ concentration (**f**) of *T. subcordiformis* cultures with different amounts of NaHCO_3_ addition (0, 0.2, 1 and 5 g L^−1^) under nitrogen depletion (mean ± SD, *n* = 3)

Nitrogen depletion was an effective strategy to induce starch accumulation in *T. subcordiformis* [5]. Therefore, the impact of NaHCO_3_ addition was investigated under nitrogen depletion. In general, microalgae can recycle the intracellular stored nitrogen (e.g., protein-derived nitrogen) to transiently support their growth when extracellular nitrogen is depleted [24]. The addition of NaHCO_3_ in the context of 2% CO_2_ aeration influenced the cell growth and biomass production under nitrogen depletion. As shown in Fig. 1a, the cell density as revealed by OD_750_ was dramatically enhanced with the addition of NaHCO_3_, which exhibited a dose-dependent manner under the NaHCO_3_ concentrations between 0 and 1 g L^−1^. The final cell density in the culture with 0.2 and 1 g L^−1^ of NaHCO_3_ addition at Day 4 was 48% and 1.1-fold higher, respectively, than that without NaHCO_3_ addition. Similarly, the biomass accumulation was also enhanced with the addition of NaHCO_3_. The maximum biomass production in the culture with 1 g L^−1^ NaHCO_3_ reached 2.6 g L^−1^ at Day 3, which was 17% and 1.1-fold higher than that in the 0.2 and 0 g L^−1^ NaHCO_3_ cultures (Fig. 1b). Noteworthily, further increasing NaHCO_3_ concentration to 5 g L^−1^ showed negative effects in terms of both cell growth and biomass production compared with the 1 g L^−1^ NaHCO_3_ culture, although it still improved biomass accumulation by 88% in comparison with the 0 g L^−1^ NaHCO_3_ culture (Fig. 1b).

The addition of NaHCO_3_ could generate a favorable pH condition for enhanced biomass accumulation in *T. subcordiformis* under nitrogen depletion. As shown in Fig. 1c, the pH of the medium generally increased evidently with the addition of NaHCO_3_, which reached average levels of 5.2, 6.7, 7.7 and 8.3, respectively, after equilibrium for one day in the cultures with 0, 0.2, 1 and 5 g L^−1^ NaHCO_3_ addition. The present study showed that the best biomass production was obtained with the addition of 1 g L^−1^ NaHCO_3_ where pH was maintained at 7.7, which was in line with the optimal pH condition for biomass production in *T. suecica* [25]. The dramatic inhibition of cell growth and biomass production in the culture without NaHCO_3_ addition could be largely ascribed to the low pH environment at around 5.2. In fact, the pH as low as 5.5 had been demonstrated to impede cell growth and reduce biomass productivity in *Tetraselmis* [25, 26]. The decrease in pH to 5–5.5 was recently shown to be the main factor contributing to the inhibition of biomass production in *Arthrospira platensis* [10], which could also apply herein. Low pH was reported to impair photosystems and inactivate some critical enzymes related to carbon assimilation (e.g., Rubisco), which caused diminished cell growth and biomass accumulation [27].

The beneficial effects of NaHCO_3_ to biomass accumulation could also be attributed to the relieving of inhibition caused by the high dissolved CO_2_ concentration (dCO_2_) in the CO_2_ aeration culture. It was evident from Fig. 1d that in the context of 2% CO_2_ aeration, no addition of NaHCO_3_ resulted in a dCO_2_ of 2.9–4.1 mmol kgSW^−1^, which was 13- to 52-fold higher than that in the cultures with 0.2 and 1 g L^−1^ NaHCO_3_ addition. Similar dCO_2_ (around 5 mmol kgSW^−1^) was also found in the cultivation of *Nannochloropsis salina* with high CO_2_ (20%) supply where biomass accumulation was strongly inhibited compared with the low CO_2_ (0.04% and 6%) supply [17]. Li et al. [13] also found that a dCO_2_ of 11.29 mM was the primary inhibitive factor for the cell growth in *C. vulgaris.* In addition, the dCO_2_ accounted for averagely 79% of the total DIC in the culture without NaHCO_3_ addition (Additional file 1: Figure S1a), further supporting the notion that the diminished biomass production could be ascribed to the inhibition caused by the high dCO_2_. The addition of NaHCO_3_ increased the total alkalinity (Additional file 1: Figure S2a) and pH (Fig. 1c) of the culture, which would convert more dissolved CO_2_ into HCO_3_^−^ according to the equilibrium relationship of CO_2_ + H_2_O ↔ HCO_3_^−^ + H^+^. As a result, the dCO_2_ was dramatically decreased (Fig. 1d). In fact, the predominant DIC in the cultures with the addition of NaHCO_3_ was HCO_3_^−^, which accounted for approximately 88%, 92% and 78% of the total DIC in the cultures with 0.2, 1 and 5 g L^−1^ NaHCO_3_, respectively (Additional file 1: Figure S1b–d). It indicated that HCO_3_^−^ was the main carbon source for the growth of *T. subcordiformis* under these conditions. HCO_3_^−^ had been shown to be an effective carbon source for *Tetraselmis* [12, 28]. Collectively, it was reasonable to conclude that cell growth and biomass production were facilitated with the addition of NaHCO_3_ via ensuring a suitable pH, alleviating inhibition of high dCO_2_ and providing sufficient effective carbon source HCO_3_^−^.

However, excessive addition of NaHCO_3_ up to 5 g L^−1^ caused unfavorable effects on cell growth and biomass production relative to the 1 g L^−1^ counterpart (Fig. 1a, b). The increased pH up to 8.3 in the 5 g L^−1^ NaHCO_3_ culture (Fig. 1c) should not be accounted for this inhibitory effect because pH ranging from 7.4 to 8.5 could not affect biomass production in *T. subcordiformis* (see the discussion in the nitrogen-limitation cultivation below). HCO_3_^−^ was the predominant DIC in both the 1 and 5 g L^−1^ NaHCO_3_ cultures, as discussed above, and thus, the different performance of *T. subcordiformis* could be reasonably ascribed to the difference in the HCO_3_^−^ concentration. It should be noted that the concentration of HCO_3_^−^ reached 26.7 mmol kgSW^−1^ in the 5 g L^−1^ NaHCO_3_ culture on Day 2 when inhibitory effects occurred, which was four times of that in the 1 g L^−1^ one (Fig. 1e). This high HCO_3_^−^ concentration in the 5 g L^−1^ NaHCO_3_ culture could be unfavorable for the growth of *T. subcordiformis*. The assimilation of HCO_3_^−^ involves an active transport in microalgae which is energy consuming and therefore bio-energetically disadvantaged [11, 13]. The excessive HCO_3_^−^ might disturb the energy supply for photosynthetic CO_2_ bio-fixation and other energy-dependent metabolism for cell growth. Therefore, superfluous addition of NaHCO_3_ caused adverse effects on biomass production.

### Photosynthetic performance under nitrogen depletion

The carbon availability and pH can impact the photosynthetic performance of microalgae, leading to varied biomass production. Therefore, several chlorophyll a fluorescence kinetics parameters were tracked throughout the cultivation to check the photosynthetic efficiency.

**Fig. 2:**
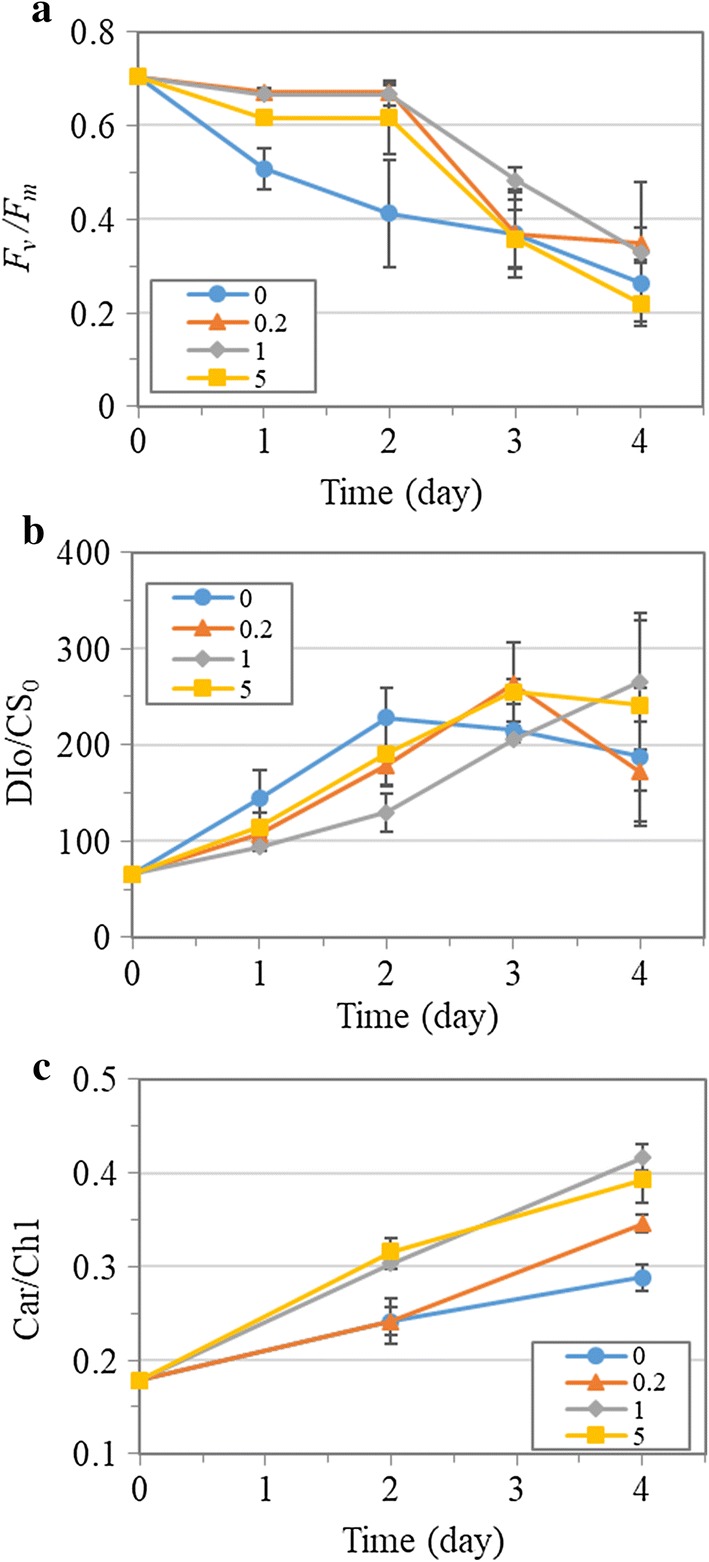
Photosynthetic activity ($F_{\text{v}} /F_{\text{m}}$, **a**), dissipated energy flux per excited cross section (DIo/CS_0_, **b**) and carotenoid/chlorophyll ratio (Car/Chl, **c**) of *T. subcordiformis* cultures with different amounts of NaHCO_3_ addition (0, 0.2, 1 and 5 g L^−1^) under nitrogen depletion (mean ± SD, *n* = 3)

$F_{\text{v}} /F_{\text{m}}$ is the maximum quantum efficiency of photosystem II and represents the photosynthetic activity, the decline of which also denotes stress conditions microalgae would have been exposed to [5]. Figure 2a shows that a sharp decline of $F_{\text{v}} /F_{\text{m}}$ (0.704 on Day 0 to 0.413 on Day 2) was present from the beginning of the cultivation in the culture without NaHCO_3_ addition, while the $F_{\text{v}} /F_{\text{m}}$ decreased slightly to 0.67 on Day 2 in the cultures with the addition of 0.2 and 1 g L^−1^ NaHCO_3_. This result indicated that NaHCO_3_ addition could alleviate the stress as well as the consequent loss of photosynthetic activity caused by the combined nitrogen depletion and low pH or high dCO_2_. Cell morphology analysis (Additional file 1: Figure S3a) on Day 2 also showed that the microalgal cells became abnormally round under nitrogen depletion without NaHCO_3_ addition, whereas it remained normally elliptical in the cultures with 0.2 and 1 g L^−1^ NaHCO_3_ addition, indicating that NaHCO_3_ addition alleviated the stress exerted on cells, which was consistent with the $F_{\text{v}} /F_{\text{m}}$ results. Nitrogen deprivation is considered to generate reactive oxygen species (ROS) in microalgae that will cause damage to the cellular organization and impair the photosynthesis [29]. It has been recently reported that NaHCO_3_ addition could reduce the oxidative stress induced by nutrient (N, P or S) deficiency and consequently improve the photosynthetic activity in *D. salina* [15], which was in agreement with the present study in *T. subcordiformis*. Furthermore, the energy dissipation flux per excited cross section (DIo/CS_0_) [30] showed an overall increase under nitrogen deprivation, with the most rapid enhancement observed in the culture without NaHCO_3_ addition and slowest with 1 g L^−1^ NaHCO_3_ addition (Fig. 2b). The promoted energy dissipation under nitrogen stress is a protective mechanism for microalgae coping with unfavorable conditions, which has also been observed in nitrogen-starved *Chlamydomonas reinhardtii* [31] and *Porphyridium cruentum* [32]. The lowest level of DIo/CS_0_ in the 1 g L^−1^ NaHCO_3_ culture during the first 2 days suggested the highest energy utilization efficiency in the microalgae and the least stress condition the microalgae were subjected to, which was in alignment with the highest photosynthetic activity ($F_{\text{v}} /F_{\text{m}}$, Fig. 2a). In addition, the carotenoid/chlorophyll ratio (Car/Chl) representing the status of photoprotective function against oxidative stress under nutrient-deprived conditions [29] exhibited continuous increase in all the cultures, and a more rapid increase was observed in the cultures with the addition of NaHCO_3_ (especially with 1 and 5 g L^−1^) relative to the non-addition one (Fig. 2c). It indicated that NaHCO_3_ addition improved the photoprotection, which could contribute to the much better photosynthetic activity therein. Overall, owing to the best maintenance of photosynthetic efficiency in the 1 g L^−1^ NaHCO_3_ culture on Day 2, the highest biomass productivity of 0.89 g L^−1^ day^−1^ and CO_2_ fixation rate of 1.67 g L^−1^ day^−1^ were achieved therein, which were 2.1-fold higher than the 0 g L^−1^ counterpart (Table 1).

**Table 1:** Biomass and starch productivity, CO_2_ bio-fixation rate, and amylose production of *T. subcordiformis* cultures with different amounts of NaHCO_3_ addition under nitrogen-depletion (−N) and nitrogen-limitation (±N) cultivation modes (mean ± SD, *n* = 3)

NaHCO_3_ (g L^−1^)	Cultivation mode	Biomass productivity (g L^−1^ day^−1^)	CO_2_ bio-fixation rate (g L^−1^ day^−1^)	Starch productivity (g L^−1^ day^−1^)	Amylose content (% total starch)	Amylose concentration (mg L^−1^)	Amylose productivity (mg L^−1^ day^−1^)
0	−N	0.29 ± 0.05^α^ (2^a^)	0.55 ± 0.10^α^ (2)	0.27 ± 0.02^α^ (2)	27.8 ± 0.3^α^ (2)	153 ± 8^α^ (2)	54 ± 4^α^ (2)
	±N	0.74 ± 0.00^A^ (8)	1.39 ± 0.00^A^ (8)	0.46 ± 0.01^A^ (8)	26.7 ± 1.5^A^ (8)	984 ± 53^A^ (8)	121 ± 7^A^ (8)
0.2	−N	0.81 ± 0.10^β^ (2)	1.52 ± 0.20^β^ (2)	0.60 ± 0.01^γ^ (2)	30.9 ± 0.5^γ^ (2)	354 ± 2^γ^ (2)	155 ± 1^γ^ (2)
	±N	0.74 ± 0.01^A^ (8)	1.38 ± 0.02^A^ (8)	0.47 ± 0.03^AB^ (8)	26.7 ± 0.9^A^ (8)	1011 ± 32^AB^ (8)	125 ± 4^AB^ (8)
1	−N	0.89 ± 0.03^β^ (2)	1.67 ± 0.05^β^ (2)	0.73 ± 0.02^δ^ (2)	32.9 ± 0.3^δ^ (2)	449 ± 16^δ^ (2)	202 ± 8^δ^ (2)
	±N	0.82 ± 0.05^B^ (8)	1.55 ± 0.10^B^ (8)	0.51 ± 0.03^B^ (8)	27.7 ± 1.0^A^ (8)	1148 ± 108^B^ (8)	142 ± 14^B^ (8)
5	−N	0.80 ± 0.15^β^ (2)	1.50 ± 0.29^β^ (2)	0.41 ± 0.12^β^ (2)	34.7 ± 0.9^β^ (2)	276 ± 68^β^ (2)	116 ± 34^β^ (2)
	±N	0.69 ± 0.05 (4)	1.29 ± 0.09 (4)	0.09 ± 0.04 (4)	59.3 ± 0.5 (4)	238 ± 83 (4)	56 ± 21 (4)

The different Greek alphabets (α, β, γ and δ) represented significant difference (*p *< 0.05) between the cultures under −N cultivation mode. The different capital Latin alphabets (A and B) represented significant difference (*p *< 0.05) between the cultures under ±N cultivation mode

^a^The number in the parentheses represented the cultivation day used for calculation and comparison

### Starch production and starch quality under nitrogen depletion

**Fig. 3:**
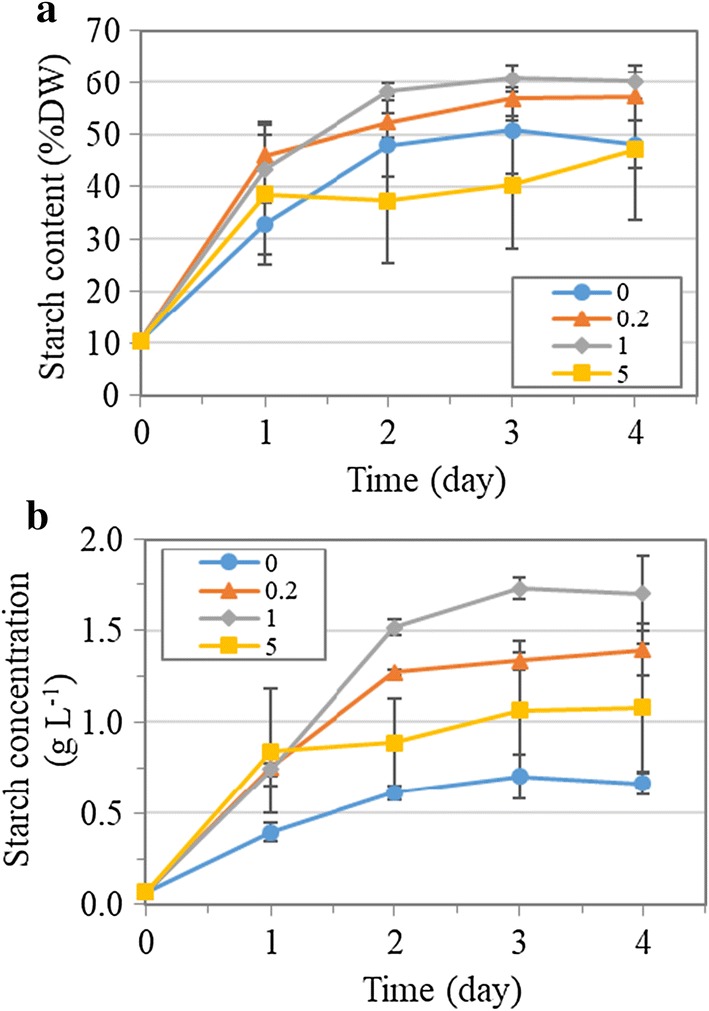
Starch content (**a**) and starch concentration (**b**) of *T. subcordiformis* cultures with different amounts of NaHCO_3_ addition (0, 0.2, 1 and 5 g L^−1^) under nitrogen depletion (mean ± SD, *n* = 3)

Starch accumulation could be stimulated under nitrogen deprivation in *T. subcordiformis*, as had been demonstrated previously [5] and here (Fig. 3a, b). It was obvious that NaHCO_3_ addition resulted in more pronounced starch accumulation under this stressful condition. The starch content increased rapidly from the initial level of 10.4%DW to the maximum of 60.6%DW and 56.9%DW within 3 days in the 1 and 0.2 g L^−1^ NaHCO_3_ cultures, respectively, while it reached only 50.7%DW in the one without NaHCO_3_ addition (Fig. 3a). As a result, the starch concentration exhibited a dose-dependent manner from the NaHCO_3_ concentrations of 0 g L^−1^ to 1 g L^−1^ (Fig. 3b). The maximal starch concentration of 1.7 g L^−1^ obtained in the culture with 1 g L^−1^ NaHCO_3_ on Day 3 was 2.5 times of that without NaHCO_3_ addition (0.7 g L^−1^). Similar to the case in the biomass production, addition of 5 g L^−1^ NaHCO_3_ to the culture led to adverse effects on starch accumulation, with the lowest starch content of 40.3%DW obtained on Day 3 therein, although the starch concentration was still superior to that without NaHCO_3_ addition due to the enhanced biomass accumulation (Fig. 3b). Starch accumulation in autotrophic microalgae relies on photosynthesis for carbon fixation and sugar-precursor (ADP-glucose) biosynthesis, both of which are energy-consuming processes [33]. Therefore, the higher photosynthetic activity under NaHCO_3_ addition, which should generate more ATP and NADPH for these two processes, could be reasonably accounted for the enhanced starch production here in *T. subcordiformis*. Moreover, the increased Car/Chl under NaHCO_3_ addition (Fig. 2c) suggested a more active cyclic electron flow around photosystem I, which could generate extra ATP in compensation for the loss of activity at photosystem II [34]. Consequently, the carbon fixation and starch accumulation could be facilitated. In addition, the varied pH itself could also be accounted for the difference of starch accumulation in *T. subcordiformis*. *Tetraselmis* sp. had been demonstrated to have lower starch content under alkaline medium (28%DW, pH 8) than those established under neutral pH (64%DW, pH 7) and under acidic medium (49%DW, pH 6), which coincided with the present study [35]. In *C. vulgaris*, starch content varied from 40 to 55% in the pH range of 6.5–9.0, with the maximum value obtained at pH of 7.7 [36], which was also in alignment with the present study. Collectively, the appropriate addition of NaHCO_3_ (e.g., 1 g L^−1^) which alleviated high dCO_2_ stress along with the formation of suitable pH environment plus oxidative stress mitigation ensured adequate photosynthesis and hence supported the starch biosynthesis. Due to the maintained photosynthetic efficiency in the first 2 days, the starch productivity peaked at 0.73 g L^−1^ day^−1^ on Day 2 in the culture with 1 g L^−1^ NaHCO_3_, which was 1.7-fold higher than the one with no NaHCO_3_ addition (Table 1).

To further reveal the influence of nitrogen depletion and NaHCO_3_ addition on the starch quality, the amylose (Am)/amylopectin (Ap) ratio (Am/Ap) was also measured. Generally, it appeared that as nitrogen deprivation prolonged, Am/Ap was enhanced in all the cultures (Table 2), indicating that amylose biosynthesis under nitrogen stress condition was more favored than amylopectin. This result coincided with the phenomenon found in *C. reinhardtii* 137C that 15–35% of amylose based on total starch (TS) was obtained under nitrogen starvation in against which < 5%TS of amylose under nitrogen repletion [37]. Nitrogen depletion had been found to stimulate granule-bound starch synthase (GBSS), a critical enzyme responsible for amylose biosynthesis in microalgae and plants [38, 39], which could also be applied in *T. subcordiformis*. Interestingly, the addition of NaHCO_3_ accelerated the increase in Am/Ap under nitrogen depletion, especially in the 1 g L^−1^ and 5 g L^−1^ NaHCO_3_ cultures (*p *< 0.05, Table 2). For example, the Am/Ap reached 0.49 (Am: 32.9%TS, Table 1) and 0.53 (Am: 34.7%TS, Table 1) in the 1 g L^−1^ and 5 g L^−1^ NaHCO_3_ cultures, respectively, on Day 2, which was 26% and 36% higher than that in the 0 g L^−1^ one (approximately 0.39, i.e., Am: 27.8%TS, Table 1). In addition, the Am content (%DW) was significantly enhanced with the increase in NaHCO_3_ addition in the concentration range of 0–1 g L^−1^ from Day 2 to Day 4 (*p *< 0.05), while the Ap content showed almost no significant difference (*p *> 0.05, Table 2), suggesting that the addition of NaHCO_3_ primarily facilitated the Am accumulation. The Am content in the culture with 1 g L^−1^ NaHCO_3_ reached 19.1%DW on Day 2, which showed 44% of improvement compared with the 0 g L^−1^ counterpart. This phenomenon, to the best of our knowledge, was rarely reported previously, the mechanism of which was poorly understood either. In fact, the enhanced Am proportion in TS was also observed in *Chlorella* under low CO_2_ (air, 0.038%) conditions where CO_2_-concentrating mechanisms (CCM) was induced to synthesize a pyrenoidal starch sheath [40, 41]. The addition of bicarbonate herein should also induce a CA-mediated CCM for carbon utilization [11]. Whether the improved amylose content should be ascribed to the CCM needs intensive study. Besides, the possibility of the influence of pH itself on amylose content could not be excluded, since low-CO_2_ cultivation always leads to increased pH [9, 17], which mimics the effect of bicarbonate addition.

**Table 2:** Am/Ap ratio, and Am or Ap content (%DW) of *T. subcordiformis* cultures with different amounts of NaHCO_3_ addition under nitrogen-depletion (−N) cultivation modes (mean ± SD, *n* = 3)

NaHCO_3_ (g L^−1^)	0				0.2				1				5			
	1 day	2 days	3 days	4 days	1 day	2 days	3 days	4 days	1 day	2 days	3 days	4 days	1 day	2 days	3 days	4 days
Am/Ap	0.41 ± 0.10^a^	0.39 ± 0.01^a^	0.46 ± 0.06^a^	0.48 ± 0.09^a^	0.44 ± 0.02^a^	0.45 ± 0.01^b^	0.48 ± 0.02^a^	0.49 ± 0.01^a^	0.46 ± 0.03^a^	0.49 ± 0.01^c^	0.58 ± 0.01^b^	0.62 ± 0.01^b^	0.46 ± 0.01^a^	0.53 ± 0.02^d^	0.63 ± 0.06^b^	0.60 ± 0.04^b^
Am (%DW)	9.6 ± 3.0^a^	13.3 ± 1.5^a^	15.8 ± 1.7^a^	15.5 ± 2.9^a^	14.1 ± 2.3^a^	16.2 ± 1.4^ab^	18.6 ± 1.4^ab^	18.8 ± 1.6^ab^	13.6 ± 2.1^a^	19.1 ± 0.6^b^	22.2 ± 1.1^b^	22.9 ± 1.2^b^	12.1 ± 3.9^a^	12.9 ± 3.8^a^	15.7 ± 5.1^a^	17.8 ± 5.6^ab^
Ap (%DW)	23.4 ± 3.5^a^	34.6 ± 4.6^b^	35.0 ± 6.9^b^	32.6 ± 3.0^ab^	31.9 ± 4.2^a^	36.2 ± 3.6^b^	38.3 ± 2.0^b^	38.5 ± 3.1^b^	29.8 ± 4.5^a^	39.0 ± 1.1^b^	38.4 ± 1.5^b^	37.2 ± 2.2^ab^	26.4 ± 9.3^a^	24.5 ± 8.2^a^	24.6 ± 7.2^a^	29.2 ± 7.7^a^

The different letters (a, b, c and d) represented significant difference (*p *< 0.05) between the cultures on the same cultivation day

### Biomass production and photosynthetic performance under nitrogen limitation

**Fig. 4:**
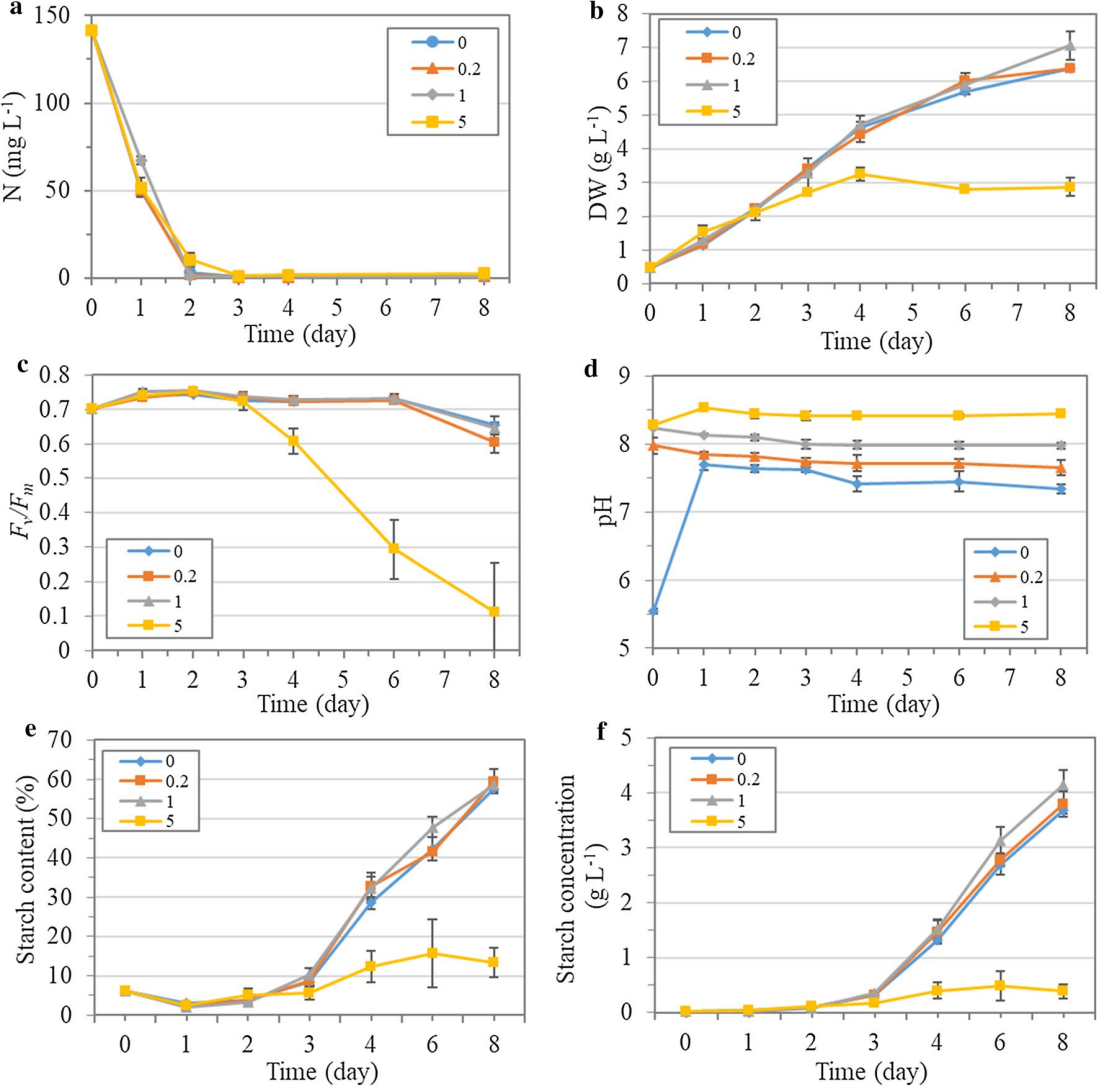
Nitrate consumption (**a**), biomass production (dry weight, **b**), photosynthetic activity ($F_{\text{v}} /F_{\text{m}}$, **c**), pH variations (**d**), starch content (**e**) and starch concentration (**f**) of *T. subcordiformis* cultures with different amounts of NaHCO_3_ addition (0, 0.2, 1 and 5 g L^−1^) under nitrogen limitation (mean ± SD, *n* = 3)

Nitrogen depletion generally led to the decline of photosynthesis and thus limited the overall biomass and starch production, although it was effective in inducing starch accumulation in microalgae. Therefore, a batch culture mode with limited nitrate supply (10 mM, nitrogen limitation) was applied in *T. subcordiformis*, in the context of which the impact of NaHCO_3_ was further evaluated. As shown in Fig. 4a, the nitrate was almost consumed up within 2 days in all the cultures, and the overall nitrate removal rate (more than 97%) exhibited no significant difference among them. The biomass accumulation showed no significant difference between the cultures with 0, 0.2 and 1 g L^−1^ NaHCO_3_ addition during the first 6 days, but significant improvement (*p *< 0.05) could be discerned on the 8th day in the 1 g L^−1^ NaHCO_3_ culture where 7.1 g L^−1^ biomass was achieved, which was 10% higher than the 0 and 0.2 g L^−1^ NaHCO_3_ cultures (Fig. 4b). The final biomass productivity and CO_2_ bio-fixation rate reached 0.82 g L^−1^ day^−1^ and 1.55 g L^−1^ day^−1^, respectively, in the 1 g L^−1^ NaHCO_3_ culture, which exhibited 11% improvement compared with the one without NaHCO_3_ addition (Table 1).

The photosynthetic activity ($F_{\text{v}} /F_{\text{m}}$) showed almost identical profile in the cultures with 0, 0.2 and 1 g L^−1^ NaHCO_3_ addition throughout the cultivation: It increased from 0.70 to 0.74–0.75 in the first 2 days when nitrate was replete, and then gradually decreased to approximately 0.63 on Day 8 with the depletion of nitrate (Fig. 4c). In addition, DIo/CS_0_ and Car/Chl showed no significant difference either in these three cultures (*p *> 0.05, Table 3). The cell morphology also suggested the little difference among the cultures with 0–1 g L^−1^ NaHCO_3_ addition (Additional file 1: Figure S3b). Collectively, it seemed that in this nitrogen-limitation batch culture mode, the addition of NaHCO_3_ up to 1 g L^−1^ in the context of 2% CO_2_ aeration exerted little impact on the photosynthesis and biomass production under the nitrogen-repletion phase and the sequential short-term (2 to 4 days) nitrogen-depletion phase. The beneficial effects of NaHCO_3_ addition on biomass production only occurred in the 1 g L^−1^ culture in the extended nitrogen-depletion phase (6 days). These results were quite different from those obtained under initial nitrogen-depletion cultivation in *T. subcordiformis* where suitable NaHCO_3_ addition (e.g., 1 g L^−1^) led to prompt and remarkable improvements of biomass production (Fig. 1b). These findings were also different from those in *Chlorella* sp. HS2 where addition of NaHCO_3_ in the context of 1% CO_2_ aeration led to a significant improvement of biomass productivity and the effect was dose dependent in the range of 0–0.75 g L^−1^ NaHCO_3_ [9]. Notably, the addition of 5 g L^−1^ NaHCO_3_ led to a dramatic inhibition of biomass accumulation and photosynthetic activity (Fig. 4b, c), as was also reflected by the enhanced energy dissipation (DIo/CS_0_) and decreased photoprotection (Car/Chl ratio) (Table 3) as well as aberrant cell morphology (Additional file 1: Figure S3b). This inhibition was much severer than that under the initial nitrogen-depletion cultivation (Fig. 1b).

**Table 3:** Dissipated energy flux per excited cross section (DIo/CS_0_), carotenoid/chlorophyll ratio (Car/Chl) and DIC (CO_2_, HCO_3_^−^ and CO_3_^2−^) concentrations in the medium of *T. subcordiformis* cultures with different amounts of NaHCO_3_ addition under nitrogen limitation (Day 8, mean ± SD, *n* = 3)

NaHCO_3_ (g L^−1^)	DIo/CS_0_	Car/Chl	CO_2_ (mmol kgSW^−1^)			HCO_3_^−^ (mmol kgSW^−1^)			CO_3_^2−^ (mmol kgSW^−1^)		
			1 day	2 days	8 days	1 day	2 days	8 days	1 day	2 days	8 days
0	125 ± 22^A^	0.301 ± 0.004^AB^	0.03 ± 0.01	0.08 ± 0.03	0.11 ± 0.02	2.54 ± 0.07	4.84 ± 1.35	3.70 ± 0.03	0.17 ± 0.03	0.27 ± 0.04	0.11 ± 0.02
0.2	120 ± 14^A^	0.302 ± 0.007^AB^	0.03 ± 0.01	0.07 ± 0.01	0.07 ± 0.01	2.90 ± 0.49	6.60 ± 0.20	4.87 ± 0.61	0.27 ± 0.05	0.57 ± 0.05	0.29 ± 0.09
1	95 ± 24^A^	0.311 ± 0.006^B^	0.03 ± 0.00	0.04 ± 0.02	0.07 ± 0.01	6.73 ± 0.30	8.36 ± 3.57	10.18 ± 0.73	1.24 ± 0.06	1.38 ± 0.53	1.28 ± 0.15
5	352 ± 87^B^	0.295 ± 0.005^A^	0.03 ± 0.01	0.06 ± 0.01	0.06 ± 0.01	16.17 ± 2.30	24.74 ± 1.61	26.29 ± 2.86	8.36 ± 0.92	10.21 ± 1.35	10.76 ± 0.80

The different capital Latin alphabets (A and B) represented significant difference (*p *< 0.05) between the cultures

The pH of the medium under nitrogen limitation reached averagely 7.5, 7.7, 8.0 and 8.4 from Day 1 to Day 8 with 0, 0.2, 1 and 5 g L^−1^ NaHCO_3_ addition, respectively (Fig. 4d), which were higher than the levels of the corresponding culture under nitrogen depletion (5.2, 6.7, 7.7 and 8.3, Fig. 1c), especially in the cultures with 0 and 0.2 g L^−1^ NaHCO_3_ addition. The pH diversity caused by the different amounts of NaHCO_3_ addition was also remarkably reduced (i.e., variation of 0.9 in nitrogen limitation vs. 3.1 in nitrogen depletion). Noteworthily, it was evident that the pH increased rapidly from 5.5 to 7.7 within 1 day in the culture without NaHCO_3_ addition (0 g L^−1^, Fig. 4d). This increase in pH could be attributed to the utilization of nitrate which led to an increase in alkalinity by releasing OH^−^ into the medium [17, 42, 43]. As a result, the increased alkalinity eliminated the potential acidification caused by the 2% CO_2_ supply, making a favorable pH environment for biomass production. Meanwhile, the elevated pH reduced the dCO_2_ and increased the HCO_3_^−^ availability as discussed above, and thus, the high-CO_2_ inhibition could be removed with simultaneously adequate effective carbon source (HCO_3_^−^) becoming available. In fact, the HCO_3_^−^ concentration reached 3.70 mmol kgSW^−1^ during the 8-day cultivation and it accounted for 94% of the DIC in the culture with 0 g L^−1^ NaHCO_3_ addition (Table 3, Additional file 1: Figure S1e), which could sufficiently support biomass accumulation. In a similar way, the elevated alkalization of medium in the cultures with 0.2, 1 and 5 g L^−1^ NaHCO_3_ addition related to their nitrogen-depletion counterparts could also be attributed to the supply of nitrate. Therefore, due to the inherent alkalization nature of nitrate uptake process in microalgae, the combined supply of nitrate and CO_2_ reduced the pH diversity and made the addition of NaHCO_3_ less useful in promoting biomass production and CO_2_ bio-fixation. It should be noted that the biomass production was almost identical in all the cultures before Day 2 and in the cultures with 0, 0.2 and 1 g L^−1^ NaHCO_3_ addition before Day 6 (Fig. 4b) where the pH was at 7.4–8.5 (Fig. 4d), indicating that *T. subcordiformis* had a relatively broad suitable pH range. This result was inconsistent with other *Tetraselmis* species such as *T. suecica* [25] and *Tetraselmis* sp. [26] where biomass accumulation varied with the pH changing at 7.5–8.5. The insensitivity of *T. subcordiformis* toward pH at this range is preferable in large-scale cultivation since the biomass productivity would be less affected when exposed to pH variations, which needs less strict pH control.

Although nitrate supply minimized the pH-regulation effect of NaHCO_3_ addition, the biomass production and CO_2_ bio-fixation could still be facilitated with the addition of 1 g L^−1^ NaHCO_3_ in the late phase (Fig. 4b). This beneficial effect might be ascribed to the relatively more abundant HCO_3_^−^ in the medium as the effective carbon source. As shown in Table 3, the HCO_3_^−^ concentration reached 10.18 mmol kgSW^−1^ in the culture with the addition of 1 g L^−1^ NaHCO_3_ on Day 8, which was 1.8- and 1.1-fold higher than that in the 0 and 0.2 g L^−1^ NaHCO_3_ counterparts. The occurrence of this advantage only in the late phase of cultivation could be due to the more need of carbon source that 1 g L^−1^ NaHCO_3_ addition was able to more easily meet when cell density reached a high level at that phase. However, excessive NaHCO_3_ addition up to 5 g L^−1^ caused an overall inhibition on photosynthesis and algal biomass production (Fig. 4b, c), and the inhibitory effects were more remarkable compared with the nitrogen-depletion culture (Fig. 1b). The enhanced pH up to 8.5 was not the reason for this inhibition, as discussed above. It was obvious that the HCO_3_^−^ increased up to 26.29 mmol kgSW^−1^ until Day 8 (Table 3), which was comparable to that under nitrogen-depletion culture (Fig. 1e). Therefore, although high HCO_3_^−^ concentration was unfavorable to biomass production here, it could not account for the elevated inhibitory effects in the nitrogen-limitation culture relative to the nitrogen-depletion one. It should be noted that CO_3_^2−^ accounted for more than 29% of DIC in the culture with 5 g L^−1^ NaHCO_3_ addition under nitrogen-limitation culture (Additional file 1: Figure S1h), and it reached more than 10 mmol kgSW^−1^ on Day 2 (Table 3), which was 1.5 times of that under nitrogen-depletion culture in the same NaHCO_3_ condition (Fig. 1f). Taken together, it could be speculated that high CO_3_^2−^, rather than HCO_3_^−^ or pH, led to the severe inhibition of photosynthesis and biomass production under nitrogen-limitation culture. CO_3_^2−^ is generally not a carbon source for microalgae due to the lack of membrane transportation system [13]. However, CO_3_^2−^ had been found to act as a strong inhibitor to HCO_3_^−^ assimilation in algae [44]. As a result, excessive CO_3_^2−^ could give rise to carbon limitation, leading to the impaired photosynthesis and biomass production. The enhanced CO_3_^2−^ could also be derived from the additional alkalization of medium as a consequence of nitrate uptake. The proportion of CO_3_^2−^ in the total DIC was highly sensitive to pH variations in the range of 8–8.5 in seawater system (salinity of 36 kg m^−3^), as demonstrated by Chen et al. [17]. The increase in pH from 8.2 in the nitrogen-depletion culture (Fig. 1c) to 8.4 in the nitrogen-limitation one (Fig. 4d) should cause a considerable enhancement of CO_3_^2−^ concentration. Therefore, the nitrate supply aggravated the inhibitory effects of high NaHCO_3_ addition.

### Starch production and starch quality under nitrogen limitation

The starch accumulation occurred after the nitrate was exhausted on Day 2, and the final starch content reached approximately 58.6% on Day 8 in the cultures with 0, 0.2 and 1 g L^−1^ NaHCO_3_ addition with no significant difference observed (*p *> 0.05), indicating that starch accumulation was unaffected with the addition of NaHCO_3_ within this concentration range (Fig. 4e). It could be due to the relatively small variations in pH (7.5–8.0) in these cultures, as was found in *C. vulgaris* under a similar pH range [36]. As a result, the starch concentration exhibited a similar profile to biomass production where significant difference could only be discerned on the final day of cultivation. The final starch concentration and starch productivity reached 4.1 g L^−1^ and 0.51 g L^−1^ day^−1^, respectively, in the culture with 1 g L^−1^ NaHCO_3_ addition, which was 12% and 11% higher than the 0 g L^−1^ NaHCO_3_ counterpart (Fig. 4f, Table 1). Like the case in the biomass production, the starch accumulation was severely inhibited in the culture with 5 g L^−1^ NaHCO_3_ addition, with a maximum starch content of only 15.6%DW and starch concentration of 0.5 g L^−1^ obtained on Day 6 (Fig. 4e).

Interestingly, the Am/Ap showed an increase from 0.17 on Day 2 to 0.65 on Day 3 and gradually decreased to 0.37 on Day 8 during the starch accumulation phase in the cultures with 0, 0.2 and 1 g L^−1^ NaHCO_3_ addition without any significant difference (*p *> 0.05, Table 4), which was quite different from the case in the nitrogen-depletion cultures where Am/Ap exhibited a continuous increase and NaHCO_3_ addition accelerated this increase (Table 2). The transient increase in Am/Ap at the start of nitrogen depletion coincided with the findings in the initial nitrogen-depletion culture (Table 2) and other microalgae such as *C. reinhardtii* 137C [37]. However, the subsequent decrease in Am/Ap was unanticipated. The most probable reason was the enhanced cell density in the nitrogen-limitation culture (3.4–7.1 g L^−1^ biomass from Day 3 to Day 8, Fig. 4b) relative to the nitrogen-depletion one (maximum of 2.6 g L^−1^ biomass on Day 3, Fig. 1b) that caused a decreased light availability because of the self-shading effects [6]. In fact, the stimulation of GBSS was shown to be light dependent [39], and low light intensity resulted in decreased GBSS activity and relative amylose content in rice [45]. Therefore, in the nitrogen-limitation culture mode, the enhanced biomass production was unfavorable for amylose production in *T. subcordiformis*. The disappeared stimulation effects of NaHCO_3_ addition on amylose production might be due to the reduced diversity of pH and HCO_3_^−^ concentration among the cultures with different NaHCO_3_ addition that stemmed from the nitrate uptake (as discussed above). Unexpectedly, the Am/Ap increased dramatically from 0.32 on Day 2 to 1.46 (Am: 59.3%TS, Table 1) on Day 4 in the culture with 5 g L^−1^ NaHCO_3_ addition (Table 4), although a weak overall starch accumulation (5.0%DW to 12.3%DW) could be observed (Fig. 4e). It was evident that the Am content increased by 5.6 times during this period, whereas the Ap content increased by only 28% (Table 4), which indicated that Ap accumulation was largely inhibited under this high-NaHCO_3_ environment, which generated a relatively high Am/Ap.

**Table 4:** Am/Ap ratio, and Am or Ap content (%DW) of *T. subcordiformis* cultures with different amounts of NaHCO_3_ addition under nitrogen-limitation (±N) cultivation modes (mean ± SD, *n* = 3)

NaHCO_3_ (g L^−1^)	0				0.2				1				5			
	2 days	3 days	4 days	8 days	2 days	3 days	4 days	8 days	2 days	3 days	4 days	8 days	2 days	3 days	4 days	8 days
Am/Ap	0.15 ± 0.03^a^	0.57 ± 0.03^a^	0.55 ± 0.16^a^	0.36 ± 0.03^b^	0.19 ± 0.03^a^	0.70 ± 0.13^a^	0.47 ± 0.04^a^	0.36 ± 0.02^b^	0.16 ± 0.02^a^	0.68 ± 0.06^a^	0.48 ± 0.05^a^	0.38 ± 0.02^b^	0.32 ± 0.13^b^	0.78 ± 0.47^a^	1.46 ± 0.03^b^	0.23 ± 0.08^a^
Am (%DW)	0.5 ± 0.2^a^	3.1 ± 0.5^ab^	10.0 ± 1.5^ab^	15.4 ± 0.8^b^	0.6 ± 0.0^a^	3.6 ± 0.9^b^	10.4 ± 0.5^b^	15.9 ± 0.4^b^	0.5 ± 0.2^a^	4.1 ± 0.9^b^	10.4 ± 0.3^b^	16.2 ± 0.6^b^	1.1 ± 0.2^b^	2.2 ± 0.3^a^	7.3 ± 2.4^a^	2.6 ± 1.4^a^
Ap (%DW)	3.4 ± 0.8^a^	5.4 ± 0.6^b^	18.6 ± 2.9^b^	42.3 ± 1.4^b^	3.2 ± 0.2^a^	5.0 ± 0.3^ab^	22.4 ± 2.8^b^	43.6 ± 2.8^b^	2.9 ± 0.9^a^	6.0 ± 0.8^b^	21.7 ± 2.9^b^	42.3 ± 1.6^b^	3.9 ± 1.8^a^	3.5 ± 1.7^a^	5.0 ± 1.7^a^	10.7 ± 2.4^a^

The different letters (a, b, c and d) represented significant difference (*p *< 0.05) between the cultures on the same cultivation day

### Choice of cultivation strategy for different purposes

The present study demonstrated that the addition of 1 g L^−1^ NaHCO_3_ in the context of 2% CO_2_ aeration was preferable under both the nitrogen-depletion (−N) and nitrogen-limitation (±N) cultivation modes in terms of biomass production, CO_2_ bio-fixation and overall starch production. The biomass productivity of around 0.86 g L^−1^ day^−1^ and starch content of 58.3%DW were almost the same under these two cultivation modes, and they exceeded most of the photoautotrophic microalgae under nutrient depletion (Table 5). The highest starch productivity of 0.73 g L^−1^ day^−1^ was obtained in the −N culture on Day 2, which was comparable to that in *Chlorella* sp. AE10 [8, 46], the best microalgal starch producer hitherto to the best of our knowledge, under photoautotrophic conditions in nitrogen depletion conditions, and the starch concentration of 1.52 g L^−1^ in *T. subcordiformis* was even higher, although biomass productivity and starch content were slightly lower (Table 5). These results demonstrated *T. subcordiformis* to be a good candidate for photosynthetic CO_2_ bio-fixation and starch production.

**Table 5:** Biomass and carbohydrate (starch, glycogen or total carbohydrate) production in microalgae under different carbon sources and nutrient stress conditions reported in literatures

Strain	Carbon source			Nutrient stress	Biomass productivity (g L^−1^ day^−1^)	Starch concentration (g L^−1^)	Starch productivity (g L^−1^ day^−1^)	Starch content (%DW)	References
	CO_2_ (%)	NaHCO_3_ (g L^−1^)	Organic carbon						
*Chlorella sorokiniana*	2	0	0	−N	0.45 (2^a^)	–^b^	0.17 (2)	38 (2)	[18]
*Chlamydomonas reinhardtii*	0.04	4.2	0	−N	–	0.79 (4)	0.18 (4)	69.3 (4)	[64]
	0.04	0	0	−N	–	0.04 (4)	–	7.3 (4)	
	5	0	0	−N	–	0.06 (4)	–	12.5 (4)	
*Chlorella vulgaris* CCALA 924	2	0	0	−N	0.23 (0.5)	0.10 (0.5)	0.19 (0.5)	37 (0.5)	[6]
	2	0	Urea (1.1 g L^−1^)	−P	0.75 (0.75)	0.35 (0.75)	0.48 (0.75)	53 (0.75)	
	2	0	Urea (1.1 g L^−1^)	−S	1.10 (0.83)	0.62 (0.83)	0.74 (0.83)	60 (0.83)	
*Chlorella* sp. AE10	10	0.016	0	−N	1.20 (2)	1.42 (2)	0.71 (2)	56.9 (2)	[46]
				−N (0.375 mM)	0.95 (2)	1.21 (2)	0.73 (2)	60.5 (2)	[8]
*Scenedesmus obliquus* CNW-N	2.5	0	0	±N (4 mM)	0.55	1.88^c^ (7)	0.27^c^ (7)	49.4^c^ (7)	[65]
*Synechococcus* sp. PCC 7002	2	168	0	±N (15 mM)	1.0 (7)	3.5^d^ (7)	0.50^d^ (7)	49.8^d^ (7)	[66]
*Arthrospira platensis*	0.04	16.8	0	±N (3 mM)	0.46 (3.5)	1.03^d^ (3.5)	0.29^d^ (3.5)	65^d^ (3.5)	[67]
*Tetraselmis subcordiformis*	2	1	0	−N	0.89 (2)	1.52 (2)	0.73 (2)	58.1 (2)	This study
	2	1	0	±N (10 mM)	0.82 (8)	4.14 (8)	0.51 (8)	58.6 (8)	

^a^The number in the parentheses represented the cultivation day used for calculation and comparison

^b^Data unavailable

^c^Data represented the total carbohydrate

^d^Data represented the glycogen from cyanobacteria

**Fig. 5:**
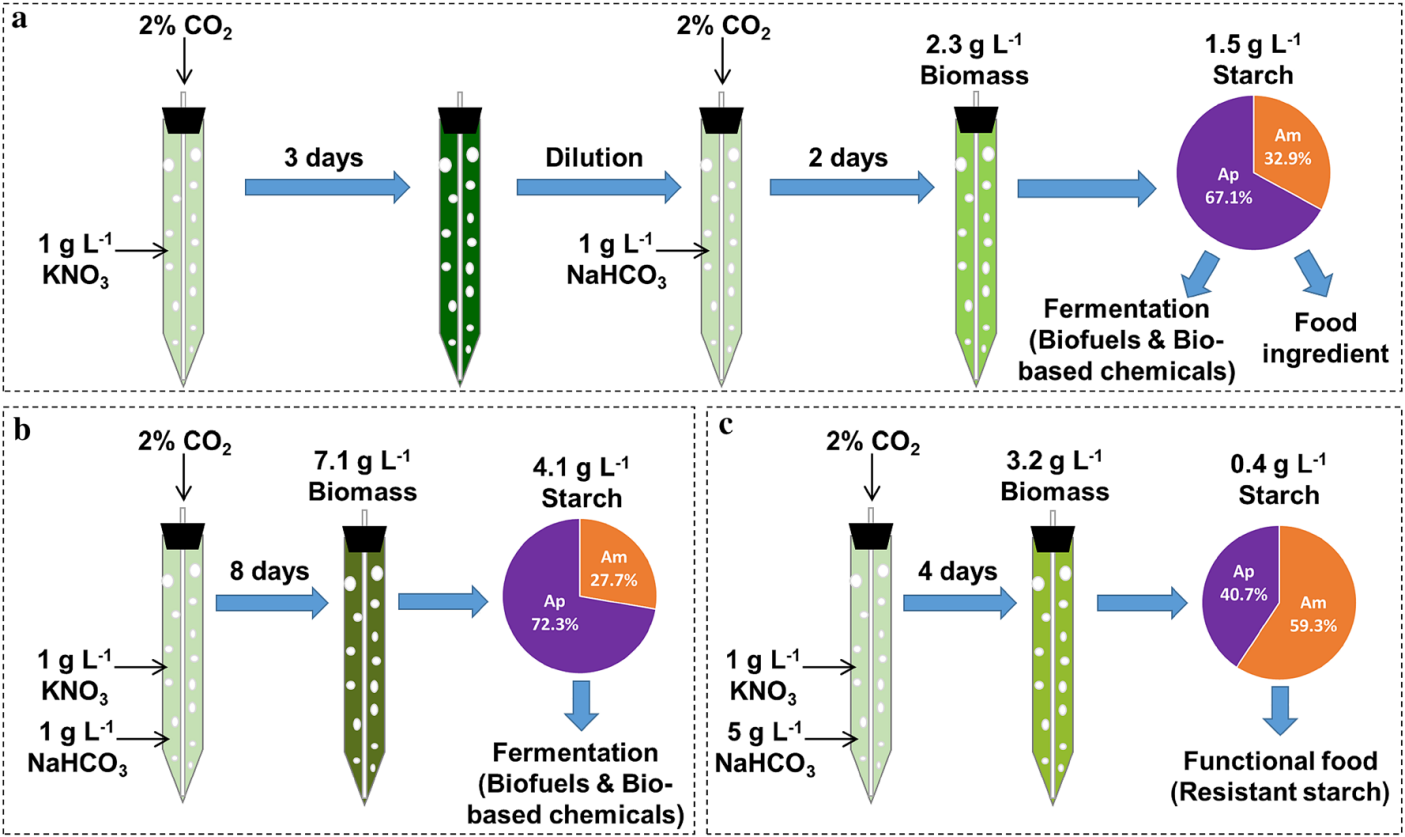
Proposed cultivation strategies for starch production in *T. subcordiformis* with diverse applications. The +N → −N strategy (**a**) comprised a +N (1 g L^−1^ KNO_3_) without NaHCO_3_ addition in the first stage and a dilution for −N with 1 g L^−1^ NaHCO_3_ addition in the second stage for short-term cultivation (2 days). The ±N strategy employed a simultaneous supply of 1 g L^−1^ KNO_3_ and 1 g L^−1^ NaHCO_3_ (**b**) or 5 g L^−1^ NaHCO_3_ (**c**) for long- or medium-term cultivation (8 or 4 days, respectively)

Considering that the biomass production was insensitive to NaHCO_3_ addition (Fig. 4b), the following cultivation strategy including a sequential transformation from nitrogen repletion (+N) to –N (+N → −N) can be proposed (Fig. 5a): The algae are first inoculated with low cell density (0.5 g L^−1^) under +N (10 mM nitrate) without NaHCO_3_ addition; after 3 days when nitrogen is completely exhausted, the algae are diluted to the initial cell density (0.5 g L^−1^) as under +N but with nitrogen-free medium containing 1 g L^−1^ NaHCO_3_ for starch production. The present study demonstrated that −N along with NaHCO_3_ addition could not only improve the total starch production, but also enhance the amylose accumulation. The amylose content (19.1%DW) and amylose concentration (449 mg L^−1^) under −N on Day 2 in the culture with the addition of 1 g L^−1^ NaHCO_3_ (Tables 1 and 2) were 2.4- to 3.5-fold of those obtained in *Chlorella sorokiniana* [41]. The amylose content in total starch reached 33%, which was comparable to *C. reinhardtii* under nitrogen deprivation with mixotrophic cultivations, or 18% higher than *Chlorella* with photoautotrophic cultivations [37, 40]. This amylose level was even higher than most of the starch from native cereal crops where amylose accounts for about 15–32% of storage starch [47]. Amylose, which is less branched and has high gelatinization temperatures than amylopectin, has been regarded as excellent food ingredients [48]. More importantly, it has been demonstrated to be resistant to digestion and therefore is regarded as one of the contributors to resistant starch that functions for the prevention and control of colon cancer, diabetes and obesity [49]. These potential high-valued applications of amylose will contribute to the economic viability of starch-based biofuel (e.g., fermentation for liquid fuels) production. Therefore, the +N → −N cultivation strategy (two-stage mode) could be more promising from the biorefinery perspective (Fig. 5a).

For ±N cultivation, the most prominent feature was the high starch concentration (4.14 g L^−1^). Compared with other microalgae reported, it was one of the highest levels among the cultures under ±N cultivation strategy (Table 5). Notably, the ±N cultivation led to a relatively low amylose content with finally 27.7%TS achieved (Table 1). This character, in contrast to the −N cultivation mode, should be more advantageous for starch-based biofuel generation because the lower amylose content in microalgal starch had been shown to have higher enzymatic hydrolysis efficiency for glucose release [41], which could improve the carbon utilization efficiency in the subsequent fermentation process. Therefore, it is more advisable to employ the ±N cultivation strategy (batch mode, Fig. 5b) where limited nitrate (10 mM) along with 1 g L^−1^ NaHCO_3_ is supplied if fermentation efficiency was the primary target.

Moreover, the unexpected high amylose content of 59.3%TS was also achieved in the culture with 5 g L^−1^ NaHCO_3_ addition under ±N cultivation mode (Table 1, Fig. 5c). This type of starch could be considered as high-amylose starch (more than 50% amylose), which could absolutely serve as a functional food for providing slowly digestible and resistant starch to reduce the glycaemia level in the human body [48, 50]. The present study exhibited the potential of producing high-amylose starch in microalgae simply through the manipulation of cultivation conditions, although at present it could only be achieved at the expense of the overall starch productivity (Table 1). Currently, the high-amylose starches are largely produced from the genetically modified (including transgenic or non-transgenic) cereal crops [47, 51], which may raise GMO issues for food. The production of high-amylose starch from microalga *T. subcordiformis* here had initiated a novel, simple and safe way, which needs further optimization.

### Preliminary techno-economic assessment of different bicarbonate and nitrogen supply strategies

To have a clearer picture of the economic potential of these different NaHCO_3_ and nitrogen supply strategies, the costs ($ kg^−1^ biomass) derived from the carbon source (CO_2_ and NaHCO_3_) and nitrogen source (KNO_3_) were evaluated. In addition, the biomass value ($ kg^−1^ biomass) based on the starch quality (i.e., amylose percentage of total starch) was assessed as well. A parameter, economic index (EI) which estimated the biomass value per unit of carbon and nitrogen costs herein, was introduced to partially reveal the economy. As shown in Table 6, under −N strategy, the cost of carbon and nitrogen source to produce 1 kg biomass was reduced by 62%, 57% and 5%, respectively, when supplying 0.2, 1 and 5 g L^−1^ of NaHCO_3_ compared with the culture without NaHCO_3_ addition, probably due to the significant improved biomass productivity. In the meantime, the biomass value in terms of starch contained increased by 1.6-, 2.8- and 2.0-fold, respectively. As a consequence, the EIs obtained by adding NaHCO_3_ were 3.2- to 8.9-fold of that without NaHCO_3_ addition, demonstrating the considerable improvements in economy. For ±N strategy, the cost of carbon and nitrogen source was reduced by only 4% in the culture with 1 g L^−1^ of NaHCO_3_ addition, and it even nearly doubled with the addition of 5 g L^−1^ of NaHCO_3_ (Table 6) due to the declined biomass productivity compared with the ±N culture without NaHCO_3_ addition (Table 1). However, in contrast, the biomass value was enhanced by 4.7-fold in the 5 g L^−1^ NaHCO_3_ culture because of the substantial increase in amylose percentage from 26.7%TS to 59.3%TS, which consequently promoted the EI by almost twice relative to the 0 g L^−1^ NaHCO_3_ one (Table 6). It thus exemplified the great contribution of producing high-valued starch (high-amylose starch) to the economy of microalgal starch production. Collectively, these results further highlighted the beneficial effects of adding NaHCO_3_ on the economic feasibility of starch production in *T. subcordiformis* under both −N and ±N cultivation modes.

**Table 6:** The estimation of the cost required for carbon and nitrogen source used for 1 kg biomass production of *T. subcordiformis* and the potential value of biomass based on different types of starch with different amounts of NaHCO_3_ addition under nitrogen-depletion (−N) and nitrogen-limitation (±N) cultivation modes

Cultivation mode	NaHCO_3_ (g L^−1^)	KNO_3_ (g L^−1^)	Culture time (day)	Starch (%DW)	Am (%TS)	Estimated starch price ($ kg^−1^)	Culture volume needed (L kg^−1^ biomass)	Cost of carbon and nitrogen source ($ kg^−1^ biomass)	Potential value based on starch ($ kg^−1^ biomass)	EI [biomass value/(C+N source cost)]
−N	0	0	2	47.9	27.8	0.43	1724	1.07	0.21	0.19
	0.2	0	2	52.4	30.9	1.01	617	0.41	0.53	1.30
	1	0	2	58.1	32.9	1.37	562	0.46	0.80	1.72
	5	0	2	37.4	34.5	1.67	625	1.01	0.62	0.62
±N	0	1.01	8	57.7	26.7	0.23	169	0.58	0.13	0.23
	0.2	1.01	8	59.5	26.7	0.24	169	0.58	0.14	0.24
	1	1.01	8	58.5	27.7	0.42	152	0.55	0.24	0.44
	5	1.01	4	12.3	59.3	6.23	362	1.15	0.77	0.67

The economic index (EI) estimated from the biomass value per carbon and nitrogen cost was introduced to evaluate the relative economic viability for scale-up

Comparing the two cultivation modes, it seemed that the addition of NaHCO_3_ under −N was generally more favorable than the ±N mode from the economic perspective. Particularly, the highest EI (1.72) was obtained under −N with the addition of 1 g L^−1^ NaHCO_3_, which should be mainly ascribed to the relatively low nutrient (carbon and nitrogen source) cost due to the high biomass productivity (Tables 1 and 6). It is believed that improvement of photosynthetic efficiency to get enhanced productivity is the key to reduce the cost and promote economic viability of large-scale microalgal biomass production [52, 53]. In addition, the higher starch quality (32.9%TS of amylose with 1 g L^−1^ NaHCO_3_) under −N relative to the ±N counterparts (~ 27%TS of amylose) further enhanced the economic viability. Moreover, the −N cultivation mode involved much less cultivation time (2 days) than the ±N one (generally 8 days), which could significantly reduce the probability of being contaminated or preyed in large-scale cultivation [54]. However, the biomass concentration in −N culture with 1 g L^−1^ NaHCO_3_ (2.2 g L^−1^) was 67% lower than the ±N counterpart (7.1 g L^−1^) (Fig. 5), which could enhance the cost of downstream processing, especially harvesting [55]. Nevertheless, the starch-enriched *T. subcordiformis* under nitrogen depletion conditions was in fact very apt to settle (unpublished data), which should largely reduce the harvesting cost [56] and minimize the negative impact on the relative scalability under −N cultivation mode. Overall, the −N cultivation with 1 g L^−1^ NaHCO_3_ addition could have the best scalability among all the conditions tested.

The present study demonstrated the potential of regulating amylose accumulation by acting on simple cultivation parameters such as NaHCO_3_ addition and nitrogen supply. Of particular interest was the production of high amylose because of its high value that could contribute to the economic feasibility of the microalgae cultivation and biorefinery, as analyzed above. However, the biomass productivity and total starch content were relatively low (Table 1, Fig. 4e). The cultivation conditions should be further optimized to get as much biomass and starch as possible to improve the scalability.

## Conclusions

The CO_2_–bicarbonate system was crucial to ensure a suitable pH, alleviate the high-dCO_2_ inhibition, and provide sufficient effective carbon source HCO_3_^−^ for the maintenance of adequate photosynthetic efficiency and increase in photoprotection to get improved biomass and starch production as well as enhanced relative amylose content in the microalga *T. subcordiformis* under nitrogen-depletion cultivation. The biomass productivity was enhanced by 2.1-fold, and the starch productivity and concentration were both improved by more than 1.5-fold in the culture with the addition of 1 g L^−1^ NaHCO_3_ compared with the one without NaHCO_3_ addition in the context of 2% CO_2_ aeration. The amylose content was also increased by 44% under this CO_2_–bicarbonate system compared to the single use of CO_2_. The +N → −N cultivation strategy (two-stage mode) could achieve high starch productivity with enhanced amylose content that was suitable for both biofuel and high-valued food production in a biorefinery scenario, whereas ±N cultivation strategy (batch mode) could get a high starch concentration and low amylose content that was promising for biofuel generation via fermentation. High-amylose starch could be produced via the addition of 5 g L^−1^ NaHCO_3_ under ±N cultivation mode in *T. subcordiformis*, which represented a new way for the production of starch-based functional food. Considering the relatively high biomass and starch productivity as well as amylose content in *T. subcordiformis*, it could be anticipated that this excellent starch-producing microalga, as a potential substitute for agricultural crops, would play an important role in the CO_2_ mitigation for the biofuel, bio-based chemicals and functional food generation in the future.

## Methods

### Algal strain and culture condition

*Tetraselmis subcordiformis* FACHB-1751 was isolated from the Huanghai Sea near Dalian, Liaoning Province, China, and maintained by the Freshwater Algae Culture Collection of the Institute of Hydrobiology (FACHB collection), Chinese Academy of Sciences. The microalgae were previously cultivated in artificial seawater (ASW) [5] with extra additions of 0.81 g L^−1^ Tris and 0.33 mL L^−1^ glacial acetic acid. Algal cells were harvested during the late exponential phase and washed twice with nitrogen-free artificial seawater (ASW-N) where nitrate was eliminated. For nitrogen-depletion (−N) cultivation, the washed cells were inoculated with OD_750_ = 0.6 in ASW-N with the addition of NaHCO_3_ to final concentrations of 0, 0.2, 1 and 5 g L^−1^, respectively. For nitrogen-limitation (±N) cultivation, an extra of 10 mM KNO_3_ was added into the medium above.

The cells were cultivated photoautotrophically in a 600-mL glass bubble column photobioreactor (50 mm diameter, 400 mm height) with a working volume of 500 mL as descried by Yao et al. [23]. An aeration of 0.4 vvm with air containing 2% CO_2_ at 25 ± 2 °C was applied to the cultures. Continuous illumination from one side with cool white fluorescent lamps that provided an incident light intensity of 150 μmol m^−1^ s^−1^ was supplied. All the experiments were done in three biological replicates.

### pH and growth measurement

Medium pH was measured using a standard bench top pH meter (ARK, pHS-4C^+^, Sichuan, China). The cell growth was determined as the optical density of the culture at 750 nm on a spectrophotometer (AOE, UV/Vis A-360, Shanghai, China). The cell dry weight (DW, g L^−1^) was determined gravimetrically according to Yao et al. [23]. Biomass productivity (*P*_b_, g L^−1^ day^−1^) was calculated as follows:

1 $$P_{\text{b}} = \frac{{{\text{DW}}_{t} - {\text{DW}}_{0} }}{t}$$

where DW_*t*_ and DW_0_ are the cell dry weight at culture times *t* and 0, respectively.

### Photosynthetic performance analysis

The photosynthetic performance with regard to the photosystem II (PS II) maximum photochemical efficiency ($F_{\text{v}} /F_{\text{m}}$) and dissipated energy flux per excited cross section (at *t* = 0) (DIo/CS_0_) were evaluated with chlorophyll a fluorescence determined using a chlorophyll fluorometer Os30p^+^ (Opti-sciences, USA). The parameters, $F_{\text{v}} /F_{\text{m}}$ and DIo/CS_0_, were calculated according to Strasser et al. [30] as follows:

2 $$F_{\text{v}} /F_{\text{m}} = (F_{\text{m}} - F_{0} )/F_{\text{m}}$$

3 $${\text{DIo}}/{\text{CS}}_{0} = F_{0} (1 - F_{\text{v}} /F_{\text{m}} )$$

where *F*_v_ represents the variation of chlorophyll fluorescence between maximal fluorescence (*F*_m_) induced by saturating pulse and initial fluorescence (*F*_0_).

### Estimates of dissolved inorganic carbon species and nitrate

Dissolved inorganic carbon (DIC) species [dissolved carbon dioxide (CO_2_ (*aq*)), bicarbonate (HCO_3_^−^) and carbonate (CO_3_^2−^)] were calculated using the software CO_2_calc [57]. The input data included: total alkalinity (TA), pH, temperature (T), pressure (P), and salinity (S). Total alkalinity (TA) was determined by acid–base titration in a seawater system according to Dickson et al. [58]. Salinity was estimated according to the composition of medium considering the amount of sodium bicarbonate and potassium nitrate under different systems, with the values ranging at 4.19–4.69. The CO_2_ constants were taken from Millero [59] based on seawater scale. The nitrate concentration in the medium was tracked using an optical method described by Chi et al. [60].

### Biochemical composition analysis

The pigments were extracted from a 1–5 mg algal pellet by ethanol and measured as described by Yao et al. [7]. The starch was extracted from alga by 30% perchloric acid and measured by sulfuric acid–anthrone method [23]. Starch productivity (*P*_s_, g L^−1^ day^−1^) was calculated as follows:

4 $$P_{\text{s}} = \frac{{{\text{DW}}_{t} C_{\text{st}} - {\text{DW}}_{0} C_{{{\text{s}}0}} }}{t}$$

where *C*_st_ and *C*_s0_ are the starch content at culture times *t* and 0, respectively.

Amylose/amylopectin ratio (Am/Ap) was determined according to Hovenkamp-Hermelink et al. [61] with minor modifications. Briefly, the starch was extracted from alga by 30% perchloric acid. After staining with a diluted (1:2, v/v) Lugol’s 1_2_–KI solution, absorbancies at 618 and 550 nm were measured. The Am/Ap was estimated from the ratio of the absorbancies by a graph in which the specific absorptions of the two compounds were introduced. Amylose content in total starch (*C*_am/ts_, %TS), amylose content in dry weight (*C*_am/dw_, %DW), amylose concentration (*C*_am_, mg L^−1^) and amylose productivity (*P*_am_, mg L^−1^ day^−1^) were calculated using the following equations:

5 $$C_{\text{am/ts}} = \frac{\text{1}}{{\text{1} + \text{1/(Am/Ap)}}}$$

6 $$C_{\text{am/dw}} = C_{\text{am/ts}} \times C_{\text{s}} \times \text{100}$$

7 $$C_{\text{am}} = {\text{DW}} \times C_{\text{am/dw}} \text{/100} \times 1 0 0 0$$

8 $$P_{\text{am}} = \frac{{C_{{{\text{am}}(t)}} - C_{{{\text{am}}(0)}} }}{t}$$

### Preliminary techno-economic assessment of different bicarbonate and nitrogen supply strategies

The cost of carbon source (CO_2_ and NaHCO_3_) and nitrogen source (KNO_3_) to produce 1 kg of biomass at laboratory production scale was calculated according to Nayak [9]. Culture volume needed to produce 1 kg of biomass (*V*, L kg^−1^ biomass) was calculated as follows:

9 $$V = \frac{{\text{1000}}}{{P_{\text{s}} \times t}}$$

The amounts of CO_2_ consumed for 1 L culture ($Q_{{{\text{CO}}_{2} }}$, kg L^−1^) was calculated according to the following equation:

10 $$Q_{{\text{CO}_{\text{2}} }} = \frac{{W_{{\text{CO}_{\text{2}} }} \times F \times {\text{Mr}}_{{\text{CO}_{\text{2}} }} \times \text{60} \times \text{24} \times t}}{{\text{24.5} \times \text{1000}}}$$

where $$W_{{{\text{CO}}_{2} }}$$ (%) was the CO_2_ concentration in the air (2%), *F* (vvm) was the aeration rate (0.4 vvm), $${\text{Mr}}_{{{\text{CO}}_{2} }}$$ (g mol^−1^) was the relative molecular mass of CO_2_ (44 g mol^−1^) and 24.5 was the molar volume of gas (L mol^−1^) at 25 °C (298.15 K). The cost of carbon source and nitrogen source for 1 kg of biomass (*C*_C+N_, $ kg^−1^) was calculated according to the following equation:

11 $$C_{\text{C + N}} = \, (P_{{{\text{CO}}_{ 2} }} \times Q_{{{\text{CO}}_{ 2} }} + P_{{{\text{NaHCO}}_{ 3} }} \times Q_{{{\text{NaHCO}}_{ 3} }} + P_{{{\text{KNO}}_{ 3} }} \times Q_{{{\text{KNO}}_{ 3} }} ) \, \times V$$

where $$P_{{{\text{CO}}_{2} }}$$, $$P_{{{\text{NaHCO}}_{ 3} }}$$ and $$P_{{{\text{KNO}}_{ 3} }}$$ represented the price of CO_2_ (0.015 $ kg^−1^ [9]), NaHCO_3_ (0.2 $ kg^−1^ [9]) and KNO_3_ (0.93 $ kg^−1^ [62]), respectively, and $$Q_{{{\text{NaHCO}}_{ 3} }}$$ and $$Q_{{{\text{KNO}}_{ 3} }}$$ represented the amounts of NaHCO_3_ and KNO_3_ consumed for 1 L culture (kg L^−1^), respectively. The biomass value (*V*_b_, $ kg^−1^ biomass) based on starch quality (i.e., amylose percentage of total starch) was assessed as follows:

12 $$V_{\text{b}} = \, P_{\text{sta}} \times C_{\text{st}}$$

where *P*_sta_ was the price of starch ($ kg^−1^ starch). Since *P*_sta_ is significantly affected by Am/Ap [63], it was assumed that *P*_sta_ was proportional to the amylose percentage of total starch (*C*_am/ts_, %TS). The *P*_sta_ was then estimated by extrapolation from the price of normal starch (0.29 $ kg^−1^ for *C*_am/ts_ of 27%TS) and high-amylose starch (6.35 $ kg^−1^ for *C*_am/ts_ of 60%TS) according to the *C*_am/ts_ obtained under different cultivation conditions (Table 1). The economic index (EI) was defined as the biomass value per unit of carbon and nitrogen costs to partially reveal the economy:

13 $${\text{EI}} = V_{\text{b}} /C_{\text{C + N}}$$

### Statistical analysis

Results are expressed as mean ± SD from three independent experiments. SPSS 16.0 software (SPSS Inc., Chicago, IL, USA) was used to perform the statistical analysis. Multiple group comparisons were performed using one-way analysis of variance (ANOVA) and Fisher’s LSD. Values of *p* < 0.05 were defined as statistically significant.

## Additional file

**Additional file 1: Figure S1.** The DIC species distribution of *T. subcordiformis* cultures. **Figure S2.** The total alkalinity (TA) of *T. subcordiformis* cultures. **Figure S3.** Cell morphology of *T. subcordiformis* cultures with different amounts of NaHCO_3_ addition under nitrogen depletion and nitrogen limitation.

## Data Availability

All data generated or analyzed during this study are included in this published article and its Additional file 1.
