# Supplementary material for: Application of an in situ CO2–bicarbonate system under nitrogen depletion to improve photosynthetic biomass and starch production and regulate amylose accumulation in a marine green microalga Tetraselmis subcordiformis
Source: Biotechnol Biofuels. 2019 Jul 16;12:184. doi: 10.1186/s13068-019-1523-7 (PMC6631860; doi:10.1186/s13068-019-1523-7)
Supplement: Supplementary file 1 — Additional file 1: Figure S1. The DIC species distribution of T. subcordiformis cultures. Figure S2. The total alkalinity (TA) of T. subcordiformis cultures. Figure S3. Cell morphology of T. subcordiformis cultures with different amounts of NaHCO3 addition under nitrogen depletion and nitrogen limitation. [file 13068_2019_1523_MOESM1_ESM.docx]

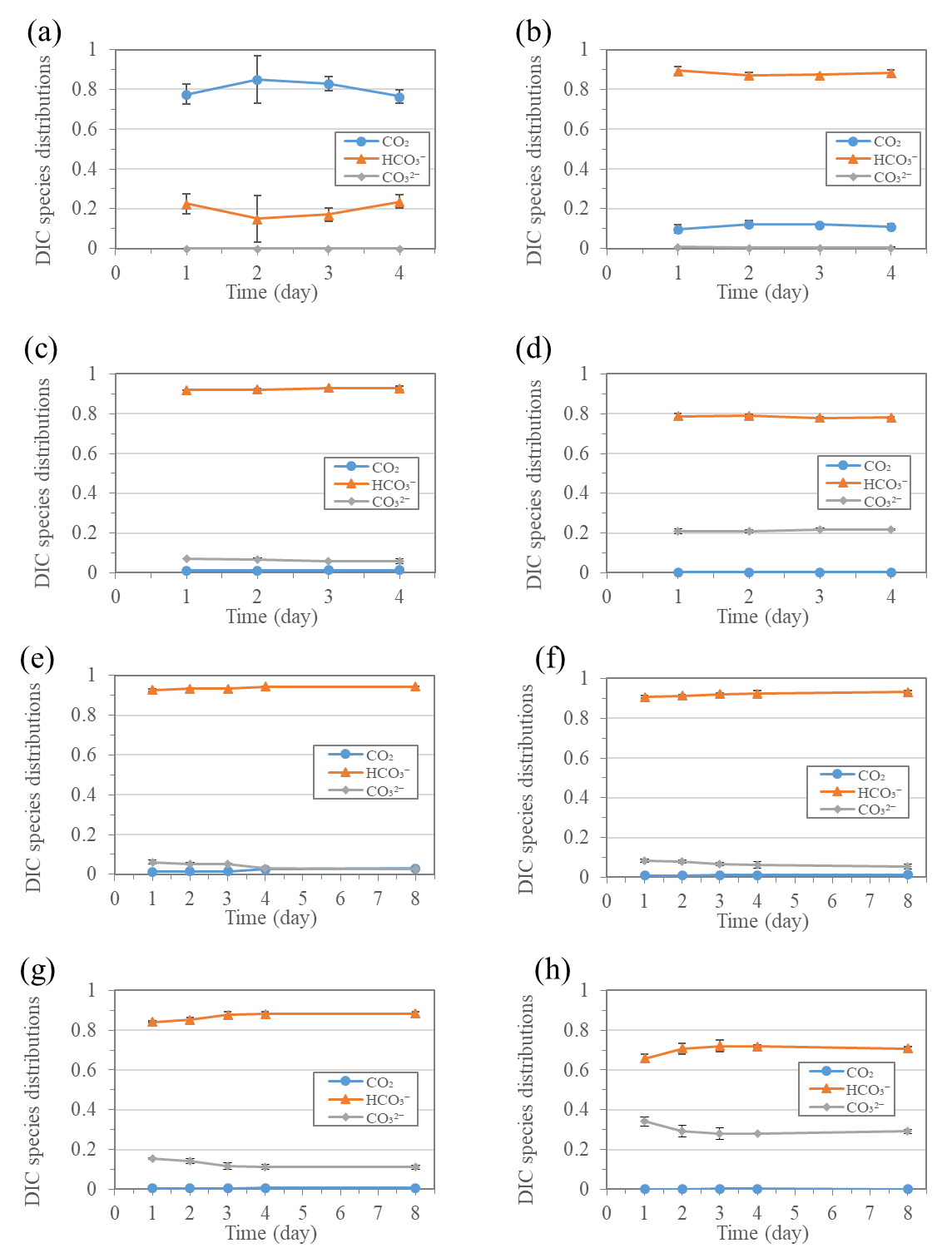


**Figure S1** The DIC species distribution of *T. subcordiformis* cultures with 0 g L^-1^ (a and e), 0.2 g L^-1^ (b and f), 1 g L^-1^ (c and g), and 5 g L^-1^ (d and h) of NaHCO_3_ addition under nitrogen depletion (a-d) and nitrogen limitation (e-h), (means±SD, n=3).


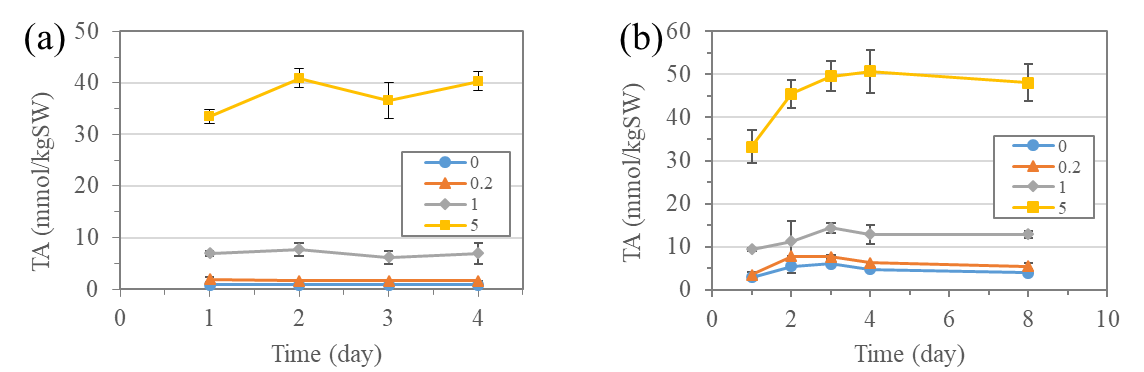


**Figure S2** The total alkalinity (TA) of *T. subcordiformis* cultures with different amounts of NaHCO_3_ addition (0, 0.2, 1, and 5 g L^-1^) under nitrogen depletion (a) and nitrogen limitation (b), (means±SD, n=3).


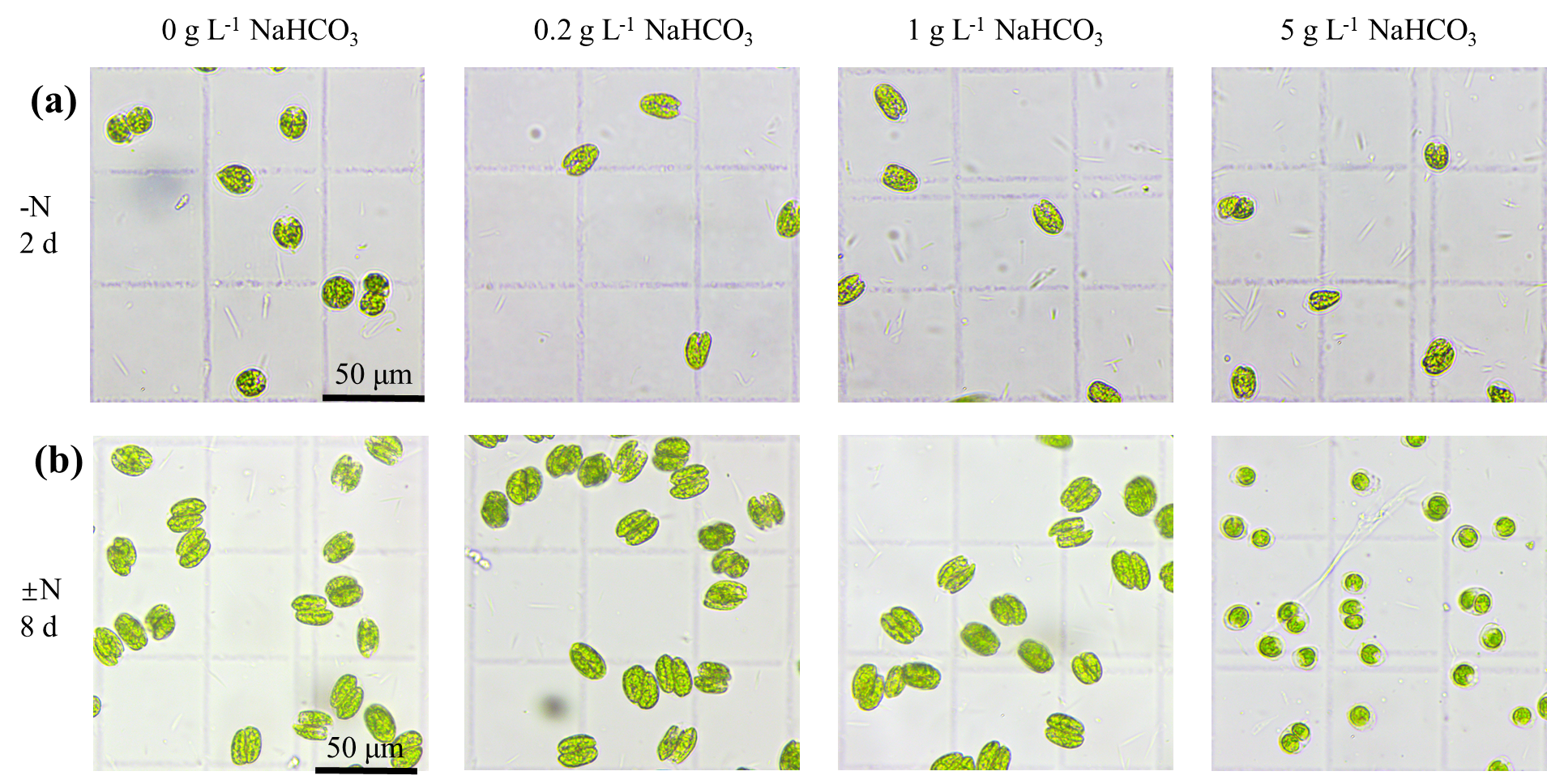


**Figure S3** Cell morphology of *T. subcordiformis* cultures with different amounts of NaHCO_3_ addition (0, 0.2, 1, and 5 g L^-1^) under nitrogen depletion (-N, a) and nitrogen limitation (b).
